# Improving responses to depression and related disorders: evaluation of a innovative, general, mental health care workers training program

**DOI:** 10.1186/1752-4458-4-25

**Published:** 2010-09-08

**Authors:** Annette L Graham, John Julian, Graham Meadows

**Affiliations:** 1 Faculty of Medicine, Nursing and Health Sciences, Department of Psychological Medicine, Southern Synergy, Monash University, Notting Hill Campus, Clayton, Vic, 3800, Australia

## Abstract

**Background:**

Australian General Practitioners have been beneficiaries of extensive training in mental health care delivery over the last few years but less so other workers who support those with mental illness. Training is needed as it is widely recognised that the most effective interventions to prevent and treat mental disorders are often not readily available. The Mental Health Aptitudes into Practice (MAP) training package is a broad, innovative, interdisciplinary, general mental health training aimed at improving responses to individuals with depression and related disorders. The modular structure of this training program meant that such training could be targeted at those with varied backgrounds. Two hundred and seventy one days of free MAP training was delivered across Victoria in 2004/2005. The evaluation reported here assessed whether changes occurred in the trainees' confidence, mental health literacy, attitudes towards effective treatments, mental health knowledge and skills and community mental health ideology following training.

**Methods:**

These elements were assessed using pen and paper tests prior, immediately following, 6 months after and then 12 months after the training. Trainees' confidence, mental health literacy and social distance were measured using scales that have been used in evaluations of Mental Health First Aid Training. Community mental health ideology was measured using a sub-scale of the Community Attitudes to the Mentally Ill (CAMI) scale. The trainees' knowledge and skills were accessed using instrumentation specifically designed for this evaluation.

**Results:**

Following training, participants had more confidence in their ability to work with those who have mental health issues and less desire for social distance from them. Participants' knowledge and skills in relation to the treatment of mental disorders increased. These changes were observed immediately after training. The limited existing evidence suggests these changes were sustained six and twelve months later.

**Conclusions:**

MAP training can be used to develop the capacity and capabilities of mental health workers.

## Background

### The need for additional training of mental health staff

Mental health services are often less than ideal. They are limited by an "efficacy-effectiveness gap (EEG) [1]." That is, the real-world outcomes of intervention are much worse than can be achieved under optimal conditions. Only a minority of those with mental illness receive treatment [2]. Even in Australia, which is reputed to have good mental health services, just over a third (35%) of those with a mental disorder in previous twelve months accessed mental health services [3]. The result of both poor treatment and under treatment of mental illness is that suffering that we know how to alleviate continues.

Effective training of mental health staff is necessary if improvements in mental health treatment are to occur and if efficacious interventions are to become more generally available. Recognizing this are a series of national and international mental health plans [4-7], all suggesting that greater training of the mental health work force should occur to facilitate improved treatment of mental illness. Those investing in the training of mental health care workers need to be assured that the training is effective. Therefore, a critical question is, what do we know about training outcomes? Back in 1999, the answer was, according to one review by Lambert and Gournay, very little [8]. These authors noted that mental health "training programs should be guided by and should generate evidence [8]."

### Existing literature, evaluated mental health training

Unfortunately, the literature addressing this issue still remains disorganized and hard to interpret. Using seven data bases (PsychINFO, A+ Education, ERIC, ProQuest education journals, Ovid Medline, Web of science, EMBASE.com and Meditext) 110 articles reporting evaluations of mental health training were identified. Most of the reported evaluated mental health training is specific. Its course content usually focuses on a narrow area of mental health care and is directed at specific sub-groups of the community or of the mental health workforce. There were a few exceptions to this, such as 'Mental Health First Aid Training', which is, as the name suggests, a general mental health training program directed at the general public. This has been extensively evaluated [9-11]. One other study was identified that described general mental health training for mental health workers from across different disciplines and from different work sites; it focused on child and adolescent mental health care [12].

No published evaluations of general mental health care training for those of different disciplines and from different work sites were identified. The search yielded only two review articles which were not especially helpful. One was a decade old [8] and the other had a limited focus: aggressive management training [13]. There is a clear need for some detailed systematic reviews of various aspects of evaluated mental health training.

### The need for general mental health training

A mental health training infrastructure that can be widely utilized needs to be developed to come closer to the goal of meeting the mental health needs of the community. This infrastructure is dependent upon evaluated mental health training programs. Unfortunately, Australia's mental health care services are fragmented. This fragmentation often leads to poor mental health care. This issue has been identified in numerous state and national reports on mental health services. For instance, in the first report by the Senate Select Committee on Mental Health, it was noted that "Compartmentalization of services (often metaphorically referred to as 'silos') [was] preventing effective care [14]." Mental health workers need to be encouraged out of the 'silos' they have traditionally inhabited. However, most of the current education and training mental health workers receive reinforces the existing fragmented structures. If the mental health workforce is ever to provide seamless care it is important for general mental health training to be directed at the whole mental health work force. Mental health workers need to develop a common idiom so there is reduced confusion between different sectors. Further, mental health workers from different sectors need to develop a sophisticated understanding of the nature of the services available in other sectors so as to ensure that their clients receive the best possible mix of services.

Improved mental health care has been a goal of both State and Federal Australian governments. In response to this need for generalist training the State Government of Victoria through beyondblue: The National Depression Initiative commissioned "the Mental Health Aptitudes into Practice (MAP)" training package which was trialled and delivered throughout Victoria.

### MAP training program

A substantial needs study was undertaken to inform those developing the training program [15,16]. It showed that there was widespread agreement about the need to expand, develop and support all those whose profession meant, that they played significant roles in the treatment of those with mental illness. It was decided not to focus on medically trained doctors as there was a variety of other training already available for them. There was also agreement on the need for training to be focused on roles and tasks that are common across a broad range of primary care and community-based services, such as the ability to identify people with or at risk of depression and related disorders, good referral practices and better care coordination for people with complex needs. Based on these findings, the aim of the training program was established, which was to enhance the overall capacity of the mental health care work force "to acknowledge, accept, and respond effectively to individuals with, and at risk of, depression and related disorders [15]."

MAP training was developed firstly for those whose professions were not primarily related to the treatment of mental illness but who were likely to come into contact with many who had mental illness (i.e. ministers of religion, police, personal carers), secondly for health and mental health primary care workers (i.e. community workers, psychiatric disability rehabilitation and support service workers [PDRS workers], aboriginal health workers) and finally for mental health professionals (i.e. psychologists, counsellors, social workers and nurses). To meet the diverse needs of these groups a modular training structure was developed with various modules being considered appropriate for those with different backgrounds and experiences.

Curriculum development occurred over a six month period and was followed by a trial period in which the training was piloted and refined. Modules were produced for seven content areas. Although seven training modules were offered only the core modules are evaluated. The core modules comprised: Module 1: Introduction to Mental Health and Mental Illness, Module 2: Depression: Introduction, Module 3: Anxiety and its Treatment, Module 4: Depression: Treatment, Prevention & Relapse.

MAP training was completed across Victoria between the 1st of April 2004 and the 4th of September 2005. Two hundred and seventy one days of free training were delivered to 2,043 individuals. Data pertinent to the evaluation was collected routinely as a component of the MAP training package. The structure of the evaluation was informed by the CIPP (context, input, process and product) evaluation model checklist [17]. This model was chosen as it is particularly focused on evaluating programs that aim to effect improvements that are sustainable in the long term [17].

Evaluation focused on participant changes across five domains that reflected the training aims. The five domains included confidence in dealing with someone with mental health issues, knowledge and skills in relation to mental health issues, mental health literacy, the social distance that they felt it necessary to maintain with those with mental disorders, and community mental health ideology. The last three of these, which are not self explanatory, are described further below.

"Mental health literacy refers to the knowledge and beliefs about mental disorders which aid their recognition, management and prevention [18]." It has been repeatedly argued that an impediment to people receiving adequate mental health care is the lack of mental health literacy [18-21].

"Social distance is the degree of proximity an individual is comfortable with in relation to a mentally ill target and it is recognized as a proxy measure of psychiatric stigma [22]." There has been some debate about whether increased mental health literacy leads to a rise in social distance [23].

"The community mental health ideology (CMHI) scale expresses sentiments concerning the therapeutic value of community, the effect of mental health facilities on residential neighborhoods, and the acceptance of deinstitutionalized care [24]." The scale is one of the four scales that make up the CAMI (community attitudes toward the mentally ill) [25].

It was expected that following attendance of the core MAP modules participants would have,

1. improved mental health literacy

2. increased knowledge and skills relating to mental health issues

3. improved confidence in their ability to help someone with mental health problems

4. decreased desire for social distance from those with mental illness

5. raised community mental health ideology

Further, it was expected that these changes would be sustained and observable six and twelve months after the training.

## Methods

### Study design

This study is a prospective evaluation assessing changes in confidence, mental health literacy, attitudes towards effective treatment, knowledge and skills and community mental health ideology following MAP training. These elements were assessed using pen and paper tests prior to the training and then immediately following the training. They were then reassessed 6 months and 12 months after the training. The evaluation was approved by the Monash University Standing Committee on Ethics in Research involving Humans.

### Subjects

There were 1126 participants in the MAP training program who completed the core MAP training modules. Of these 876 (77.8% of the participants) consented to be involved in this evaluation. The training was designed to be delivered to workers from diverse sectors of the mental health workforce, therefore, the evaluation focused on general skills and attitudes that could be seen to be shared by all those who are likely to work with those with mental illness rather than on specific skills required by those in various mental health professions.

### Evaluation materials

Attempts were made to collect evaluation data on four occasions from each participant: Table 1 details the extent of data obtained at each stage of the MAP evaluation. The majority of the questions in the MAP evaluation questionnaire were extracted from other mental health training evaluations or general research in mental health. The question accessing the participants' confidence was adapted from a question first reported by Kitchener and Jorm [9]. A higher score on this confidence measure indicates increased confidence. The questions assessing social distance were derived from the work of Link, Phelan, Bresnahan, Seueve and Pescosolido [26]. A lower score on this measure indicates that the trainee desired more social distance. The mental health literacy questions were first used by Goldney [18]. Taylor and Dear [25] developed four scales to measure community attitudes toward the mentally ill; community mental health ideology is one of these scales and was used in this evaluation. The higher the score on this measure the more positive the trainees' view of mental health service provision in the community was. A series of 38 questions asked participants to provide a self assessment of their knowledge and skills. The 18 knowledge questions covered the following areas: understanding of the burden of mental illness, causes of depression and other mental disorders, mental illness risk factors, mental illness treatments, mental health services and resources for the treatment of mental illness. The 20 skills questions covered the following areas: identifying those with mental illness, assessing their needs, engagement and communication skills, referral skills, ability to work collaboratively and dealing with distressed clients. The higher the trainees' score on both the knowledge and skills measure the greater their self assessed skills and knowledge.

**Table 1:** Characteristics of the MAP evaluation participants

Aspect of the MAP evaluation	Number	% of those who attended core modules	% of those who consented	Completed Long version of questionnaire	Completed Short version of questionnaire	Mean age at commencement of training (SD)	% Male
Attended the core modules	1126	-	-	-	-	-	-
Consented to be involved in MAP evaluation	876	77.8	-	-	-	-	-
Pre-test data	861	76.5	98.4	861	0	44.7 (10.5)	12.5%
Post-test data	684	60.7	78.1	684	0	44.9 (10.7)	10.7%
Post-6 month-training data	444	39.4	50.7	96	348	45.1 (10.4)	12.2%
Post-12 month-training data	337	29.9	38.5	42	295	45.8 (10.2)	13.3%

The initial evaluation questionnaire (the long version) took participants approximately 20 minutes to complete. All the participants completed this long version of the questionnaire prior to the training (pre) and immediately following their training (post). Due to concerns about the response rate a shortened version of the questionnaire was constructed. A series of factor analyses and RASCH analyses of pre and post questionnaires from the first 150 participants allowed researchers to determine which questions to retain in the shortened version of the questionnaire. The shortened version of the questionnaire took participants approximately 4 minutes to complete (details of this analysis are not yet published but can be obtained by contacting the first author of this paper). Most of those who completed the evaluation questionnaire six and then twelve months after the training completed the shortened version of the MAP evaluation questionnaire (see Table 1).

### Statistical analysis

#### Examining differences amongst those who completed different numbers of the MAP evaluation questionnaires

Differences in the ages of those who completed various numbers of questionnaires were examined using an ANOVA. The proportion of males and females completing different numbers of the evaluation questionnaires was examined using a chi-squared analysis. The data collected in the evaluation questionnaire was used to form variables that were either ordinal or continuous. To determine if there were any differences between those who completed one, two, three or four of the evaluation questionnaires on these variables in the initial questionnaire Kruskal-Wallis tests were used on the ordinal data and one way ANOVA was used on the continuous data.

#### Examining Changes over time

To maximise the amount data examined, three groups of data were examined:

1. Data from 185 people who completed all four (pre, post, post6, and post 12) of the evaluation questionnaires

2. Data from 358 people who completed the pre, post and post6 evaluation questionnaires

3. Data from the 674 people who completed the pre and post evaluation questionnaires.

Changes over time on the ordinal variables were examined using the Friedman test. Changes over time on the continuous variables were examined using repeated measures ANOVA.

## Results

### The evaluation participants

The participants' ages ranged from 18-81 years (mean = 44.7, median = 46, SD = 10.5) the majority of the participants were over forty. Close to 90% of the participants were female (Table 1).Table 2 lists the occupations of the participants and shows that the participants were drawn from varied occupations. Twenty percent of the participants classified themselves as nurses; the trainers estimate that approximately one third of these nurses were primary care nurses, one third were working in the aged care sector and one third had other roles in the community. Table 2 also describes the qualifications of the participants.

**Table 2:** The participant's occupations and qualifications

Occupation	Number	%	Occupation	Number	%
Nurse	180	20.9	Aboriginal health worker	9	1.0
Social worker	96	11.1	Physiotherapist	9	1.0
Personal carer/aged care worker	85	9.9	Childcare/child welfare	9	1.0
Welfare worker	74	8.6	Carer support workers	9	1.0
Youth worker/youth health worker	32	3.7	Administrator	8	0.9
Working in school environment	27	3.1	Dietician	8	0.9
Housing support worker	24	2.8	Volunteer	8	0.9
PDRS workers	24	2.8	Family support worker	8	0.9
Community worker	24	2.8	Health promotion worker	7	0.8
Women's health/support worker	19	2.2	Employment consultant	7	0.8
Occupational therapist	17	2.0	Police	6	0.7
Manager	15	1.7	Speech pathology	6	0.7
Counselor	13	1.5	Pastoral work	5	0.6
Case manager	13	1.5	Outreach worker	5	0.6
Psychologist	12	1.4	Respondent did not complete this question	12	1.4
Disability worker	10	1.2	Other	80	9.4
**Highest qualifications**				**Number**	**%**
Certificate I, II or year 10 or less				36	4.2
Year 11/12				58	6.7
Certificate III				36	4.2
Certificate IV (inc Div 2 nurse)				100	11.6
Div I Nurse				56	6.5
Diploma (inc associate diploma)				143	16.6
Batchelor Degree				249	28.9
Postgraduate diploma/graduate certificate				133	15.4
Masters degree				37	4.3
Doctorate				1	0.1
Other				3	0.3
Respondent did not complete this question				9	1.2

### Differences between those who completed all the evaluation measures and those who didn't

The 876 participants in the evaluation completed various combinations of the MAP questionnaires. A one way ANOVA showed that there were no significant age differences between those who completed different numbers of questionnaires F(3, 831) = 2.254 p = .081. A chi-squared analysis showed that there were no significant differences in the proportion of males and females who competed different numbers of questionnaires χ^2^(3,857) = 4.75 p = .191. A chi-squared analysis on a slightly restricted sample (those who had completed as masters degree or higher were excluded from the analysis so as not to violate the assumptions of chi-squared) showed that there was no significant difference in the educational attainment of those who completed different numbers of questionnaires χ^2^(21, 810) = 18.63 p = .609. To examine whether there were any differences in the professions of those who completed different numbers of questionnaires it was necessary to examine a restricted sample as cell sizes were too small for statistical examination of most of the professions. Only the five most prevalent professions (nurses, social worker, personal carer/aged care worker, welfare worker, and youth worker/youth health worker) were included in the analysis. There was no difference in the proportion of these professionals who completed different numbers of the questionnaires χ^2^(12, 466) = 5.23 p = .950. A series of different statistical tests (Kruskal-wallis for ordinal data and one way ANOVA for continuous variables) were conducted and showed that were no significant differences in the participants' initial confidence scores, mental health literacy scores, knowledge and skill ratings and community mental health ideology scores if they completed different numbers of questionnaires. These tests are described in Table 3.

**Table 3:** Examining differences in those who completed different number of MAP questionnaires

**Confidence in helping someone with mental health problems by number of questionnaires completed**			
*Ordinal Variables (analysis using Kruskal-Wallis test)*	*Chi-squared value*	*df*	*p*
Confidence	7.43	3	.06
			
**Mental Health Literacy Variables by number of questionnaires completed**			
*Ordinal Variables (analysis using Kruskal-Wallis test)*	*Chi-squared value*	*df*	*p*
Becoming more physically active such as playing more sport, or doing a lot more walking or gardening	3.70	3	0.30
Seeking information about mental health problems, its treatment and available services	7.58	3	0.06
Cutting out alcohol altogether	1.76	3	0.62
Psychotherapy - discussion about causes that stem from the client's past	0.08	3	1.00
Electroconvulsive therapy (ECT)	1.28	3	0.73
Taking over-the-counter medications (including herbal remedies)	3.85	3	0.28
Seeking help from a GP	7.42	3	0.06
Seeking help from a mental health professional (e.g. psychologist, psychiatrist)	0.68	3	0.88
Trying to deal with her problems on her own	5.01	3	0.17
			
**Social Distance by number of questionnaires completed**			
*Continuous variables (analysis using one way ANOVA)*	*F values*	*df*	*p*
Social distance (based on three questions shortened version of questionnaire)	3.85	3	0.77
Social distance (based on five questions long version of questionnaire)	0.03	3	0.99
			
**Mental Health Knowledge and skills measures by number of questionnaires completed**			
*Continuous variables (analysis using one way ANOVA)*	*F values*	*df*	*p*
Knowledge	0.94	3	0.42
Skills	0.78	3	0.51
			
**Community mental health ideology by number of questionnaires completed**			
*Continuous variables (analysis using one way ANOVA)*	*F values*	*df*	*p*
Community mental health ideology	.51	3	.67

### Changes following the completion of MAP training

#### Confidence

**Figure 1:**
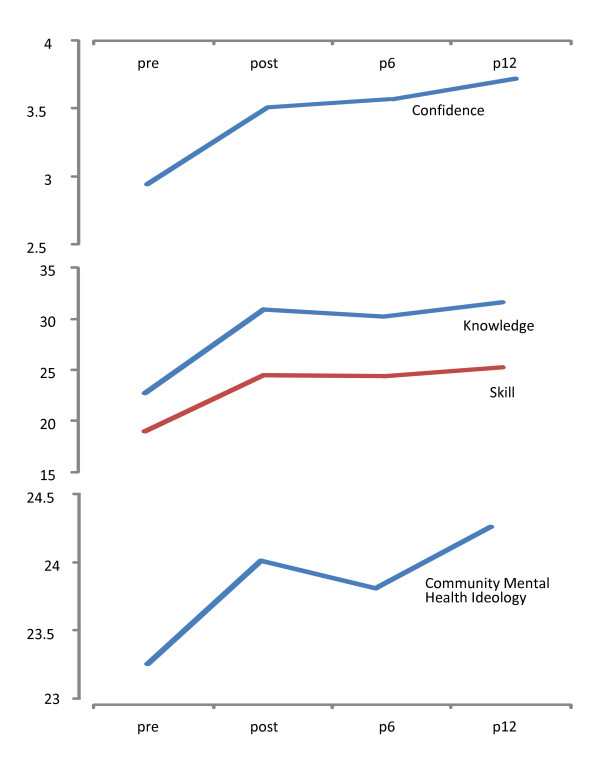
**Changes in confidence, knowledge, skills and mental health ideology immediately following MAP training**. Pre = prior to MAP training. Post = immediately after MAP training. P6 = 6 months after MAP training. P12 = 12 months after MAP training.

Participants' confidence in their ability to deal with people with mental health problems increased significantly following MAP training (see Table 4 and Figure 1). Participants maintained this increased confidence at the six and twelve month evaluation. Prior to MAP training 65.5% of the MAP participants scored three or above on this measure following MAP training 93% scored three or above on this scale.

**Table 4:** Examining changes in confidence and mental health literacy following MAP training (analysis completed using Friedman test)

Mean									
Confidence (range 1-5)	Questionnaires completed	N	pre	post	post6	post12	chisquared	Df	sig
	pre, post	653	2.83	3.49	n/a	n/a	290.04	1	< 0.000
	pre, post, post6	324	2.88	3.52	3.55	n/a	224.28	2	< 0.000
	pre, post, post6, post12	157	2.89	3.50	3.57	3.72	158.91	3	< 0.000
**Mental Health Literacy (range 1-3)**									
Becoming more physically active such as playing more sport, or doing a lot more walking or gardening	pre, post	638	2.94	2.99	n/a	n/a	16.89	1	< 0.000
	pre, post, post6	337	2.94	2.99	2.98	n/a	10.67	2	0.005
	pre, post, post6, post12	176	2.93	2.98	2.97	2.98	10.36	3	0.016
Seeking information about mental health problems, its treatment and available services	pre, post	639	2.92	2.95	n/a	n/a	4.09	1	0.043
	pre, post, post6	336	2.94	2.96	2.97	n/a	3.40	2	0.183
	pre, post, post6, post12	175	2.94	2.97	2.96	2.99	4.74	3	0.192
Cutting out alcohol altogether	pre, post	608	2.45	2.51	n/a	n/a	7.64	1	0.006
	pre, post, post6	317	2.45	2.49	2.71	n/a	52.34	2	< 0.000
	pre, post, post6, post12	164	2.46	2.47	2.74	2.73	54.19	3	< 0.000
Psychotherapy - discussion about causes that stem from the client's past	pre, post	586	2.65	2.63	n/a	n/a	0.29	1	0.592
	pre, post, post6	305	2.67	2.63	2.78	n/a	19.62	2	< 0.000
	pre, post, post6, post12	154	2.66	2.66	2.69	2.77	5.18	3	0.159
Electroconvulsive therapy (ECT)	pre, post	569	1.5	1.96	n/a	n/a	140.52	1	< 0.000
	pre, post, post6	289	1.50	1.98	1.83	n/a	87.85	2	< 0.000
	pre, post, post6, post12	141	2.01	2.84	2.57	2.59	56.29	3	< 0.000
Taking over-the-counter medications (including herbal remedies	pre, post	597	2.15	2.06	n/a	n/a	9.17	1	0.002
	pre, post, post6	307	2.19	2.05	2.06	n/a	12.06	2	0.002
	pre, post, post6, post12	155	2.15	1.98	1.99	2.01	8.42	3	0.038
Seeking help from a GP	pre, post	639	2.96	2.98	n/a	n/a	8.17	1	0.004
	pre, post, post6	338	2.97	2.99	2.99	n/a	2.13	2	0.344
	pre, post, post6, post12	176	2.99	2.99	2.99	2.98	0.39	3	0.942
Seeking help from a mental health professional (e.g. psychologist, psychiatrist)	pre, post	630	2.96	2.96	n/a	n/a	0.50	1	0.480
	pre, post, post6	329	2.95	2.97	2.98	n/a	5.30	2	0.070
	pre, post, post6, post12	170	2.95	2.98	2.98	2.98	3.79	3	0.285
Trying to deal with her problems on her own	pre, post	634	1.28	1.29	n/a	n/a	0.17	1	0.682
	pre, post, post6	329	1.30	1.29	1.35	n/a	4.84	2	0.089
	pre, post, post6, post12	163	1.25	1.26	1.34	1.37	10.41	3	0.015

#### Social distance

**Figure 2:**
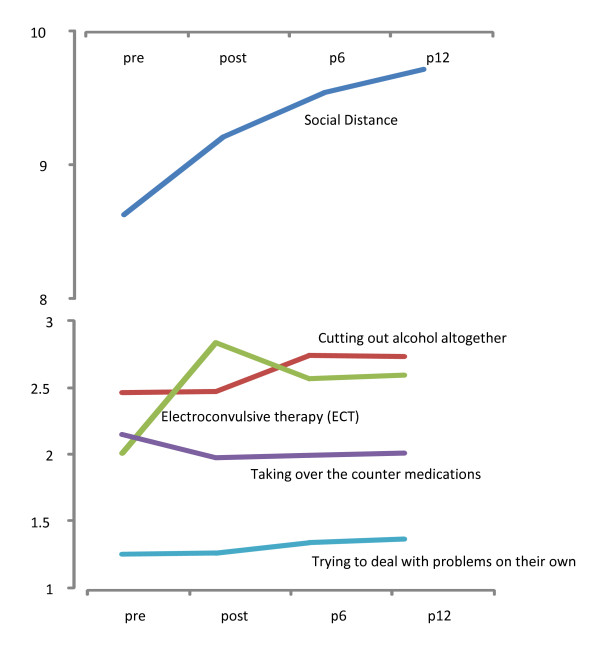
**Changes in mental health literacy and social distance immediately following MAP training**. Pre = prior to MAP training. Post = immediately after MAP training. P6 = 6 months after MAP training. P12 = 12 months after MAP training.

A higher score on the social distance measure indicated that participant was willing to be more socially engaged with a person with a mental disorder. Participants' social distance scores increased following MAP training (see Table 5 and Figure 2). These scores continued to increase in the twelve months following the MAP training.

**Table 5:** Examining changes in social distance, knowledge, skill and community mental health ideology following MAP training (analysis completed using repeated measures ANOVA)

	Questionnaires	N	Mean pre	Mean post	Mean post6	Mean post12	F	df	adjusted df	sig	partial η 2
**Social distance**	pre, post	597	8.50	8.99	n/a	n/a	54.51	1	n/a	< 0.000	.084
	pre, post, post6	312	8.58	9.07	9.46	n/a	60.86	2	n/a	< 0.000	.131
	pre, post, post6, post12	158	8.63	9.21	9.54	9.72	27.06	3	2.66	< 0.000	.147
**Knowledge**	pre, post	616	22.48	30.71	n/a	n/a	1453.04	1	n/a	< 0.000	.703
	pre, post, post6	311	23.00	31.02	30.29	n/a	471.69	2	1.95	< 0.000	.603
	pre, post, post6, post12	154	22.68	30.86	30.21	31.62	224.35	3	2.88	< 0.000	.595
**Skill**	pre, post	629	18.64	24.30	n/a	n/a	989.16	1	n/a	< 0.000	.612
	pre, post, post6	320	19.23	24.56	24.47	n/a	336.67	2	1.92	< 0.000	.513
	pre, post, post6, post12	165	18.98	24.41	24.33	25.26	166.02	3	2.79	< 0.000	.503
**Community mental health ideology**	pre, post	609	23.00	23.57	n/a	n/a	22.85	1	n/a	< 0.000	.036
	pre, post, post6	329	23.07	23.75	23.63	n/a	7.78	2	1.95	0.001	.023
	pre, post, post6, post12	169	23.25	24.01	23.81	24.26	5.72	3	2.90	0.001	.033

#### Mental health literacy

Participants' attitudes to nine different treatments for mental disorders were sought in this evaluation. Table 4 details how the participants' understanding of the helpfulness of these treatments changed or remained the same following MAP training. There was almost no change in participants' attitudes to five treatments: first, seeking help from a GP, second, seeking help from a mental health professional, third, seeking information about mental health problems, fourth, becoming more physically active and finally psychotherapy. Participants believed that these treatments were helpful prior to MAP training commencing and their attitude towards these did not change following MAP training.

There were changes following MAP training in participants' attitudes to four treatments. These are depicted in Figure 2. The treatment that MAP training elicited the greatest change in understanding about was electroconvulsive therapy. Following MAP training participants tended to believe that electroconvulsive therapy was more helpful than they had prior to their training. Following training participants also believed that cutting out alcohol altogether was more helpful than they had prior to training. Participants' attitudes to a person trying to deal with problems on their own changed marginally following training - this was believed to be slightly more helpful than it was before MAP training. Following training there was a decrease in how helpful participants believed that taking over the counter (non-prescription) medications would be for those with a mental disorder.

#### Knowledge and skills

Figure 1 and Table 5 show that participants' self-reported mental health knowledge and skills increased following training and that this increase was maintained both six and twelve months after the training had ceased.

#### Community mental health ideology

There was a slight and statistically significant increase in the participants' community mental health ideology (see Table 5 and Figure 1). The statistically significant change in means that was observed when examining the data from all those who had returned pre and post questionnaire arose because 45.2% indicated more positive community mental health ideology following training, 22.2% of the samples opinions were unchanged following training and 32.6% indicated a more negative attitude.

## Discussion

In each of the five domains examined in this evaluation positive changes were observed in the trainees following MAP training. First, trainees had significantly more confidence in their ability to work with those who have mental health issues. Second, they had decreased desire for social distance from those with mental health problems. Third, their mental health literacy had improved. Fourth, participants' knowledge and skills in relation to the treatment of mental disorders increased. Finally, there was an increase in participants' community mental health ideology. The nature and the implications of the results in each of the five domains will be explored next.

### Confidence

Three papers were identified that reported the effects of mental health first aid training on confidence [9,27,28]. Each of these papers used the same confidence measure that was used in this study. Table 6 summarises the results from these three papers as well as results from the current study. The proportion of people whose confidence increased following core MAP training was greater than has been found amongst those completing mental health first aid training. It also shows that the change in the average confidence score was greater following MAP training than had previously been reported.

**Table 6:** Comparing results on the confidence scale and social distance scale with those found in other studies

Confidence				
Paper	Training received	Percentage of participants scoring >2		Range
		Prior to training	After training	
[9]	Mental Health First aid training	62.2%	83.3%	21.1%
[28]	Mental health first aid training - workforce	54.5%	74.5%	20.0%
Current	Core MAP training	65.5%	93.0%	27.5%
		**Mean score on the confidence measure**		
		**Prior to training**	**After training**	**Range**
[27]	Mental health first aid training - rural area	3.13	3.39	.27
Current	Core MAP training	2.83	3.49	.66
**Social Distance**				
**Paper**	**Subjects**	**Average score** per question*		**Range**
		**Pre**	**Post**	
[9]	Mental health first aid training participants	1.6	1.5	.1
[28]	Mental health first aid training participants- workplace Setting	1.7	1.5	.2
[27]	Mental health first aid training - rural area	1.6	1.5	.1
[29]	Mental health first aid training - in drought affected area	2.0	1.6	.4
[26]	Nationwide representative sample USA	2.25	-	-
[30]	Mental health first aid training - expatriate Chinese community	2.0	1.8	.2
Current	Participants in Map Training course	2.2**	2.0**	.2

*The higher the score the more social distance the trainee wanted from someone with a mental illness

**These scores have been reversed to facilitate comparison.

### Social Distance

Six papers were identified that utilised the same social distance measure that was used in this evaluation [9,26-30]. The results from these papers and the current paper are summarised in Table 6. The magnitude of change observed in the MAP trainees' desire for social distance from a person with depression following MAP training is comparable with that observed in the studies evaluating mental health first aid training.

One of the concerns raised about mental health education is that it may lead to greater stigmatisation of those with mental disorders. Lauber, Carlos and Wulf [23] suggest that "improving mental health literacy may increase social distance toward people with mental illness [p. 745]." While the relationship between mental health literacy and social distance has not been explored in depth in this analysis it is worth noting that for the recipients of this training their mental health literacy increased and their desire for social distance from those with mental illness decreased. Caution must be made in accessing the significance of this, as those who were trained in the MAP were mental health workers; members of the general public may react differently to mental health education.

### Mental Health Literacy

The trainees' attitudes to the majority of the treatments examined in this study remained unchanged following MAP training, at the pre and post test the trainees indicated that they believed these to be helpful interventions. These treatments were: seeking help from GP, seeking help from a mental health professional, seeking information about mental health professional, becoming more physically active and psychotherapy. It is unsurprising that those in the Victorian mental health workforce did not be need to be convinced that these were effective and helpful treatments.

There were two treatments (trying to deal with problems on their own and taking over the counter medication) where there was a statistically significant change in the trainees' attitude but the practical change in responses was small (see Figure 2). However, participants' attitudes to electroconvulsive therapy and cutting out alcohol altogether changed markedly. Participants' belief at the completion of training was that these were more beneficial for their clients. The observed changes in attitudes to electroconvulsive therapy (ECT) were considered noteworthy as this is often a negatively publicized area in mental health care. While all the issues regarding the therapeutic efficacy of ECT are still not entirely resolved it is widely used and has a growing evidence base supporting its efficacy as a valid therapeutic tool for treatment of depression, including severe and resistant forms [31].

### Knowledge and Skills

MAP participants' self assessed knowledge and skills were shown to have improved following MAP training. The knowledge and skills questions used in this evaluation were compiled specifically for this study; therefore there are no published results using these scales to allow us to contextualise the extent of the observed changes.

### Community mental health ideology

Five papers were identified that report community mental health ideology scores following use of the same scale that was used in this evaluation [24,25,32-34]. Prior to receiving any training the average score on the community mental health ideology scale of the MAP participants was 3.8. This was equal to the highest score previously published- that found by Granello and Pauley [32] when they examined the community mental health ideology of those who work with clients who have mental illnesses. Hence, the finding of this study is consistent with previous findings suggesting that those who work with the mentally ill generally have high levels of community mental health ideology.

Following MAP training trainee average score per question on the community mental health ideology scale was 4.0. This is the highest published group score on this scale that the authors could identify (see Table 7). The difference between the MAP participants' pre test scores and post test score was on average 0.2. This was equivalent to gender differences that have been reported by Talyor and Dear [25] and Hinkelman and Granello [33]. However, it is a much smaller difference than the group difference found when the effects of exposing subjects to different types of media coverage of mental health has been examined [24,32]. Care must be made in interpreting and contextualising these differences as all the previous papers examined differences in community mental health ideology scores amongst different subgroups. That is, they examined between subject differences. The current study was unique in examining within subject differences or changes over time.

**Table 7:** Comparing results on the community mental health ideology scale with those found in other studies

Paper	Subjects	Means available for	Average score per question	Range
[25]	Toronto residents	Male	2.6*	.1
		Female	2.7*	
		Married	2.6*	.2
		Widowed	2.6*	
		Divorced	2.8*	
		Separated	2.8*	
[31]	Undergraduate students at large midwestern American University	Family member mental illness	3.4	.7
		Work with those with mental illness	3.8	
		Shown -classes	3.5	
		Shown -electronic media	3.1	
		Shown- print media	3.7	
[24]	Undergraduate students from introductory psychology classes	Stigma - given fact orientated mental illness articles	3.3	.8
		Control - given two general health articles	2.5	
		Given an article that addressed misconceptions about	2.4	
		mental illness and correct information on mental illness	2.6	
		Given an article that discussed media distortions of mental illness and target article		
[32]	Undergraduate students at a large midwestern American University	Males	3.4	.2
		Females	3.6	
[33]	Canadian police offices	Police offices	2.6	-
Current	Victorian(Australian) Mental health workers attending training course	Pre-test	3.8	.2
		Post-test	4.0	

* This measure was originally scored in the opposite direction; it has been reverse scored here to facilitate comparisons.

### Limitations

While the improvements that were observed following MAP training are striking, it must be noted that this evaluation has limitations. Only self-reported changes in those who were part of the training program were examined. The changes that are of particular interest would be changes in the way trainees treated their clients and therefore changes in the way their clients experienced mental illness. A much more extensive and costly evaluation would be required to explore whether these client-centered changes occurred.

A second limitation is the lack of a control group. There is no reason to suspect that the substantial changes that were observed would have occurred as a result of current secular trends but this cannot be definitively excluded without a control group.

The third limitation of this study resulted from trainee attrition. Data was obtained from approximately 80% of the MAP trainees who agreed to participate in the evaluation immediately after the training. After period of six months data was obtained from only 50% and after a gap of a year data was obtained from approximately 40% of the participants. The proportion of people responding to the six month questionnaire in this study was low compared to other studies. Six months after training clinicians to treat borderline personality disorder Krawitz [35] obtained evaluations from 62% of his original sample, while in the Iowa depression study data was collected data from 76% of the original sample six months after the training [36], and those conducting the evaluations of mental health first aid training were able to obtain evaluation responses from 80% of their sample six months after training [9]. No comparable studies could be identified where attempts were made to collect mental health training evaluation data twelve months after the completion of the study.

Information was received during the follow-up phases of the study from the workplaces of approximately 4% of the trainees stating that the trainee no longer worked at that particular workplace. It is impossible to estimate how many other MAP trainees shifted worksites during the follow up period and were thus unable to be contacted. It is probable that there was higher workplace mobility amongst those who received MAP training (i.e. the non-medical primary mental health care workforce) than amongst the clinicians who received the personality disorder training [35] and the mental health professionals who were trained in the Iowa depression awareness recognition and treatment program [36].

A meta-analysis suggests two effective strategies that can be used to positively influence response rates to a postal survey in the health domain [37]. The first strategy is an implementation of a reminder system. It was not feasible to introduce a reminder system into the MAP evaluation due to the number of the trainees. The second strategy suggested is shortening the questionnaire. Initially the evaluation questionnaire took approximately 20 minutes to complete, as such it was a relatively long questionnaire. As the researchers became aware that this was an issue the length the questionnaire was reduced following RASCH analysis.

The data was explored to test whether there were any detectable differences between those who filled in different numbers of the questionnaires. There were no significant demographic differences and there were no differences in the variables of interest in the evaluation: confidence, mental health literacy, mental health knowledge and skills and community mental health ideology. No evidence of sample variability across the four waves of the study was detected. Still, it is possible that a source of variability did exist that was undetected. Therefore, a cautious interpretation must be made of the results from the six and twelve month follow up as it is not clear how generalisable they are.

A final limitation of this work is that we are not certain how representative those who were trained are of the mental health work force. Almost 90% of the participants in this evaluation were female and the majority of them were aged over 40. They had varied occupations and levels of academic training. The extent to which the participants are representative of the non-medical primary mental health care workforce in Victoria is unclear as no descriptive data describing this workforce could be identified.

## Conclusions

The Boston consulting group was commissioned by the Victorian Government to lay out the framework for the next wave of reform of the mental health system. In the introduction to this report the following statement appears:

"However, additional funding alone will not be enough. Success will require commitment to improving mental health workforce capacity and capabilities, and to more effective collaboration across the whole mental health system [7]."

This evaluation shows that MAP training can be used to develop the capacity and capabilities of primary care mental health workers - something that is critical if we are to improve our mental health services. Those who participated in the evaluation received significant benefits. Following training they showed improved mental health literacy, deceased desire for social distance from those with mental disorder, improved confidence in their ability to deal with mental illness and increased knowledge and skills concerning mental illness. The available evidence suggests that these improvements were still present twelve months after the training.

## Competing interests

The authors declare that they have no competing interests.

## Authors' contributions

ALG was responsible for the data collection, management and analysis; she was also responsible for the drafting and redrafting of the current paper. JJ was the senior MAP trainer, he played a significant role in the design of the training materials and in the delivery and co-ordination of the MAP training. GM was the director of the MAP project; he conceived the project and he has directed and shaped the project since its inception. All authors have read and approved the final manuscript.
